# Visual movement perception in deaf and hearing individuals

**DOI:** 10.2478/v10053-008-0131-z

**Published:** 2013-06-17

**Authors:** Nadine Hauthal, Pascale Sandmann, Stefan Debener, Jeremy D. Thorne

**Affiliations:** Department of Psychology, Carl von Ossietzky University, Oldenburg, Germany

**Keywords:** deafness, cross-modal plasticity, localization of motion, direction of motion

## Abstract

A number of studies have investigated changes in the perception of visual motion as a result of altered sensory experiences. An animal study has shown that auditory-deprived cats exhibit enhanced performance in a visual movement detection task compared to hearing cats (Lomber, Meredith, & Kral, 2010). In humans, the behavioural evidence regarding the perception of motion is less clear. The present study investigated deaf and hearing adult participants using a movement localization task and a direction of motion task employing coherently-moving and static visual dot patterns. Overall, deaf and hearing participants did not differ in their movement localization performance, although within the deaf group, a left visual field advantage was found. When discriminating the direction of motion, however, deaf participants responded faster and tended to be more accurate when detecting small differences in direction compared with the hearing controls. These results conform to the view that visual abilities are enhanced after auditory deprivation and extend previous findings regarding visual motion processing in deaf individuals.

## Introduction

Consequences of auditory deprivation have been the subject of research for several decades. It is still not fully clear, however, which functions of the remaining senses are altered with deafness, and under which conditions these changes are relevant for behaviour. Observable auditory cortex activation in response to stimulation of intact sensory modalities in deaf animals and humans supports a cross-modal plasticity hypothesis (for a recent review, see Merabet & Pascual-Leone, 2010). This concept states that the lack of information in one sense (e.g., audition) somehow instigates the deprived cortical area to process information from the intact modalities (e.g., vision and touch). In accordance with this, animal studies have shown that visual and tactile information is indeed processed in auditory areas (Meredith & Allman, 2012; Meredith et al. 2011; Meredith & Lomber, 2011). In cases of human deafness there is also enhanced processing of visual and tactile information in the auditory cortex (Auer, Bernstein, Sungkarat, & Singh, 2007; Fine, Finney, Boynton, & Dobkins, 2005; Finney, Fine, & Dobkins, 2001; Levänen, Jousmäki, & Hari, 1998). These human studies,however, have not provided direct evidence for a causal relationship between auditory cortex activation and enhanced performance in the intact modalities. Recently, a causal relationship was shown for the first time in an animal study by Lomber, Meredith, and Kral (2010) using visual motion stimuli. This study reported that deactivation of the posterior auditory cortex eliminated the former superiority of deaf over hearing cats in a peripheral visual localization task. The same held for deactivation of the dorsal auditory cortex and performance in a task measuring the detection of visual movement. Experimental cooling of these cortical areas thus very specifically eliminated superior task performance in deaf cats. The authors therefore demonstrated that specific areas within the auditory cortex were related to the enhanced visual performance of auditory deprived cats.

In human research, several studies have observed enhanced visual abilities in deaf compared with hearing individuals. In particular, they have shown that deaf participants detect light points at larger eccentricities than their hearing counterparts (Buckley, Codina, Bhardwaj, & Pascalis, 2010; Codina et al., 2011; Stevens & Neville, 2006), suggesting larger visual fields in deaf individuals. Although they used a different method in their visual localization task, Lomber et al. (2010) showed that deaf cats have superior localization abilities in the visual periphery, a result that could be considered equivalent. Previous human studies using static visual stimuli have also revealed faster performance in deaf individuals than hearing controls in detection (Bottari, Nava, Ley, & Pavani, 2010; Chen, Zhang, & Zhou, 2006; Loke & Song, 1991) and localization tasks (Dye, Hauser, & Bavelier, 2009). Consistent with the findings on deaf animals (Lomber et al., 2010), the neural substrate for these behavioural improvements in deaf humans has been associated with cross-modal reorganization of the auditory cortex. For example, previous human studies have shown activations within the auditory cortex in response to purely visual motion in deaf individuals (Fine et al., 2005; Finney et al., 2001) and to reversing chequerboard images in cochlear-implant (CI) users (Sandmann et al., 2012). Furthermore, greater recruitment of motion-selective visual areas has been found in deaf individuals (Bavelier et al., 2000, 2001). Additionally, at a much earlier stage of processing, neural changes in deaf participants’ retinal structures have been related to enhanced peripheral vision (Codina et al., 2011). Thus, behavioural changes in deaf individuals seem to be accompanied by specific retinal adaptations, greater involvement of visual motion areas, and cross-modal reorganization of the deprived auditory cortex. In terms of clinical relevance, a relationship between visual evoked potentials and the speech perception abilities of CI users has also been reported (Buckley & Tobey, 2011; Doucet, Bergeron, Lassonde, Ferron, & Lepore, 2006). Moreover, Sandmann et al. (2012) showed that in CI users the right auditory cortex was activated when passively monitoring visual stimuli. The activation was inversely related to the speech recognition ability with the CI. This finding supports that in some CI recipients, the cross-modal reorganization that took place before implantation during the period of deafness was not completely reversed by the use of the CI and may be one of the reasons for a limited clinical benefit of the device. In light of the recently found causal relationship between superior visual abilities and auditory cortex activation in deaf animals (Lomber et al., 2010), the investigation of visual abilities in deaf humans could also contribute to the understanding of why some CI users have a greater benefit than others.

Despite the descriptions of visual enhancements in deaf individuals, it is likely that not all aspects of vision are modified following early onset deafness. One problem is that task demands and stimulus material have differed across previous experiments (see Pavani & Bottari, 2011, for a recent review). Visual sensory thresholds for contrast sensitivity, for instance, are comparable in deaf and hearing individuals (Finney & Dobkins, 2001; Stevens & Neville, 2006), as is the ability to detect changes in motion velocity (Brozinsky & Bavelier, 2004). Several neuroimaging (Bavelier et al., 2000, 2001) and electrophysiological (Armstrong, Neville, Hillyard, & Mitchell, 2002; Neville & Lawson, 1987) studies have reported enhanced processing of motion in deaf compared to hearing individuals, but behavioural results relating to the direction of motion are inconsistent. Bosworth and Dobkins (1999, 2002) presented random dot patterns to deaf and hearing individuals and found no overall difference in coherence thresholds. Neville and Lawson (1987), in contrast, used an apparent motion stimulus, namely, two squares presented consecutively at adjacent positions producing an illusory movement, and reported a performance advantage for deaf participants in detecting the direction of this motion. Thus, enhancements of visual motion perception in deaf individuals might be stimulus- or task-selective, and this may be revealed only under specific conditions.

In the present study, we tested different visual functions in deaf and in normal hearing humans in order to better understand changes in visual motion perception after early onset deafness. In one experiment, participants had to discriminate a moving and a static visual dot pattern and indicate the location of the movement. This experiment aimed to show whether the ability to localize motion is enhanced in deaf individuals as was shown in deaf cats (Lomber et al., 2010). A second experiment was conducted in order to better understand changes in the ability to perceive different directions of motion. Here participants had to discriminate a horizontally and a diagonally moving dot pattern and indicate which of two dot patterns was moving diagonally. Results from this experiment should help to clarify the inconsistency in previous human direction of motion research (Bosworth & Dobkins, 1999, 2002; Neville & Lawson, 1987).

## Movement localization task

### Material and methods

#### Participants

Twenty-two deaf individuals took part in the experiment. The criterion for inclusion of the deaf participants was a binaural hearing loss of at least 90 dB hearing level for the better ear (pure-tone average at 0.5, 1, and 2 kHz). Data from two deaf participants were discarded because of problems in their vision. Data from one deaf participant could not be analysed due to very slow responses outside the valid RT window (1,900 ms). Thus, the experiment included 19 deaf participants (*M*_age_ = 44.6 years, *SD* = 8.0, 10 male, nine female). The hearing impairments were of different aetiologies. Fourteen participants were deaf since birth. The remaining five participants became deaf before the age of 7. All deaf participants were fluent users of German Sign Language (DGS) and all but one stated that they use it every day. Three participants acquired DGS from their deaf parents, another three started to learn DGS while they were at nursery school (age 3 to 4), and 10 started learning DGS at school (age 6 to 8). Another three participants acquired sign language rather late, at age 16, 18, and 24, respectively. More details for the deaf participants are listed in Table 1. Twenty-two normally hearing individuals served as a control group. These participants were required to have an average auditory threshold no worse than 20 dB hearing level (pure-tone average at 0.5, 1, and 2 kHz). This criterion led to the exclusion of one participant. Data from two more hearing participants were discarded because of problems in vision and because of regular participation in action video gaming, respectively (as enhancements in visual attention for action video game players have been reported; for a review, see Dye, Green, & Bavelier, 2009). The remaining 19 hearing controls (*M*_age_ = 45.9 years, *SD* = 8.7, 10 male, nine female) were each assigned to one deaf participant of the same sex and handedness, and similar age. None of the hearing participants had any knowledge of sign language. There was one left-handed participant in each group with all other participants being right-handed. None of the participants had a history of central nervous system damage. All participants included in the analysis had normal or corrected-to-normal vision. All participants gave informed consent and were paid for their participation. The study was carried out in accordance with the Declaration of Helsinki principles and was approved by the local ethics committee of the University of Oldenburg.

**Table 1.** Deaf Participants’ Details

Participant	Sex	Age (years)	Cause of deafness	Age at deafness onset (years)
1	M	35	Unknown	Birth
2	F	32	Genetic	Birth
3	F	51	Measles	1
4	M	55	Unknown	Diagnosed at 3-4^a^
5	M	56	Unknown	Birth
6	M	36	Maternal rubella	Birth
7	F	42	Unknown	Birth
8	M	52	Unknown	Birth
9	F	46	Vaccination	4
10	M	37	Maternal rubella	Birth
11	M	36	Genetic	Birth
12	M	44	Unknown	Birth
13	M	43	Unknown	Diagnosed at 0,5^a^
14	F	41	Unknown	Birth
15	F	41	Maternal rubella	Birth
16	F	41	Maternal rubella	Birth
17	F	60	Unknown	Birth
18	M	53	Vaccination	6
19	F	47	Genetic	Birth

*Note*. F = female. M = male. ^a^Those participants were diagnosed at the stated age, but did not know if they were able to hear before.

#### Stimuli

**Figure 1.**
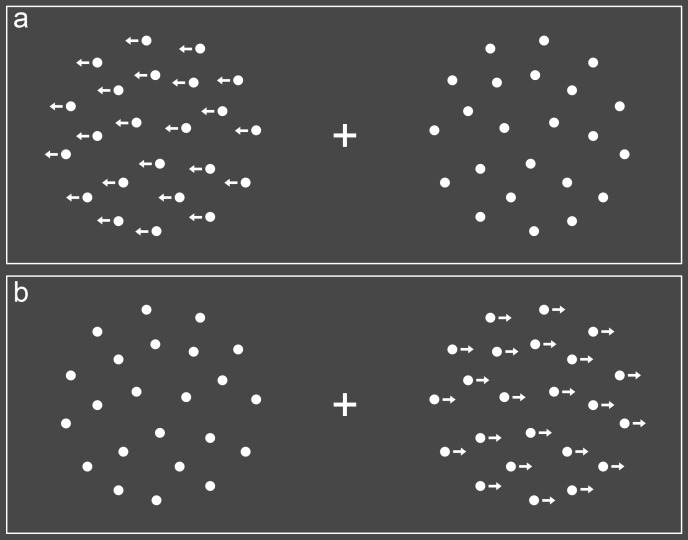
Stimuli used in the movement localization task. Trials consisted of motion either on the left side (Panel A) or on the right (Panel B). Arrows indicate the direction of motion for illustration purposes only. Dots on the opposite side remained static.

Two dot patterns were presented simultaneously left and right of a central fixation cross. Each consisted of 300 white dots (diameter = 0.01° visual angle [VA]) arranged in a circle (diameter = 3.66° VA). Stimuli were centred horizontally at an eccentricity of 6.25° VA. One dot pattern moved coherently with a speed of either 0.03°/s, 0.10°/s, or 0.15°/s. Speed levels were determined on pilot data. Movement was always horizontal. Dots in the left pattern moved leftwards and dots in the right pattern moved rightwards. In each trial, only one of the two stimuli moved, while the other was stationary (see Figure 1). The moving stimulus was presented with a probability of 50% on each side. A dot extending beyond the borders was replaced with a dot on the opposite side of the circle. White stimuli were presented on a grey background (R: 71, G: 71, B: 71).

#### Procedure

The movement localization task and the direction of motion task were run successively in one session with a short break in between. The order of tasks was counterbalanced across participants. Stimulus presentation was controlled by Presentation 14.8 software (Neurobehavioral Systems) on a personal computer, using a 61 cm monitor (1,920 × 1,080 × 32 bit colour, 60 Hz refresh rate). Participants were comfortably seated in a silent and dimly-lit room at a viewing distance of 175 cm, and held a response pad on their lap while responding with their dominant hand. In the movement localization task, participants were instructed to indicate which of the two stimuli was moving by pressing either the left button with their index finger or the right button with their middle finger. Written instructions were complemented by oral speech for hearing or sign language for deaf participants. A white fixation cross remained in the middle of the display for the whole trial and participants were told to keep their fixation during the whole experiment. At the beginning of each trial, a jittered interval of 500 to 1,000 ms elapsed before the two dot patterns appeared on the screen for 400 ms. The response window was 1,900 ms from stimulus onset. Participants were instructed to respond as fast and as accurately as possible. In sum there were 192 trials presented in randomized order, with a break after half of the trials. Participants completed 10 practice trials. Feedback was given for correct and incorrect as well as for absent responses during practice trials only.

#### Data analysis

The experimental within-subject factors were Speed and Side. The latter factor was included because several studies have reported asymmetries in movement perception in deaf and hearing individuals (Bosworth & Dobkins, 1999, 2002; Brozinsky & Bavelier, 2004; Neville & Lawson, 1987). All trials with an incorrect response, or with no response, were excluded from the analysis of response times (RTs). Median RTs were then calculated for each participant in each of the six conditions. These data were entered into a three-way mixed ANOVA, with repeated-measures factors Side (left, right) and Speed (slow, intermediate, fast), and between-subject factor Group (deaf, hearing). Greenhouse-Geisser corrected F and *p* values are reported in cases of violations of the sphericity assumption. Post hoc *t*-tests were performed where appropriate.

### Results and discussion

As expected, participants performed with near 100% accuracy (*M* = 98.8, *SD* = 1.8 %). The ANOVA on RTs revealed a main effect of speed, *F*(1.34, 48.38) = 94.52, *p* < .001. Subsequent paired-samples *t*-tests were conducted. RTs were shorter for fast (*M* = 457 ms) than for intermediate (*M* = 465 ms) and shorter for intermediate than for slow motion (*M* = 507 ms), all *ps* < .001, indicating a gradual increase in RTs with decreasing speed of motion. Thus, slow motion took longer to locate than fast motion which is in line with previous studies (Hohnsbein & Mateeff, 1992; Kreegipuu & Allik, 2007) in finding a negative relationship between velocity and detection time for motion stimuli.

**Figure 2.**
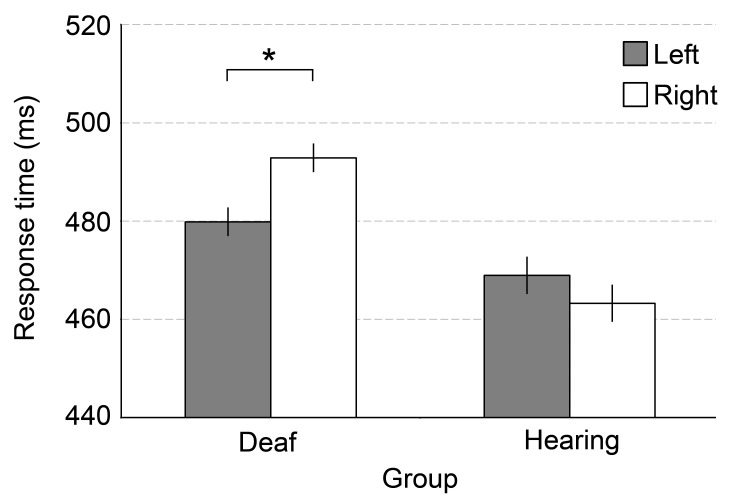
Grand mean of the participants’ median response times in milliseconds in the movement localization task for motion presented on the left and right side and for deaf and hearing participants. Error bars represent within-subject standard errors of the mean (SEM). *p < .05.

There was also an interaction between Side and Group, *F*(1, 36) = 4.16, *p* < .05 (see Figure 2). Subsequent paired-samples *t*-tests for each group showed that in hearing participants, responses for left and right sides were not significantly different (*M* = 469 vs. 463 ms), *t*(18) = 0.78, *p* = .45, whereas deaf participants were faster in locating motion on the left than on the right side (*M* = 480 vs. 493 ms), *t*(18) = 2.35, *p* < .05.

This left visual field advantage for deaf participants contradicts previous findings of a right visual field advantage for motion processing in this group (Bosworth & Dobkins, 1999, 2002; Brozinsky & Bavelier, 2004; Neville & Lawson, 1987). This apparent discrepancy will be further discussed in the General Discussion section. There was no significant main effect of Group and no Group-by-Speed interaction (all ps > .10). The lack of a significant overall difference between deaf and hearing participants’ performance in the movement localization task will also be discussed.

## Direction of motion task

### Material and methods

#### Participants

Participants were the same as in the movement localization task. However, five deaf participants did not follow task instructions and therefore performed the direction of motion task incorrectly. One participant responded to the wrong target, one did not respond to conditions 3° and 6°, and three participants responded at chance level over all angle conditions, resulting in a mean accuracy more than two standard deviations below the mean. These deaf participants as well as their matched hearing counterparts were excluded from the analysis. Thus 14 deaf (*M*_age_ = 43.3 years, *SD* = 7.8; nine males, five females) and 14 hearing participants (*M*_age_ = 45.1 years, *SD* = 9.0; nine males, five females) were analysed. There was one left-handed participant in each group with all other participants being right-handed.

#### Design and stimuli

**Figure 3.**
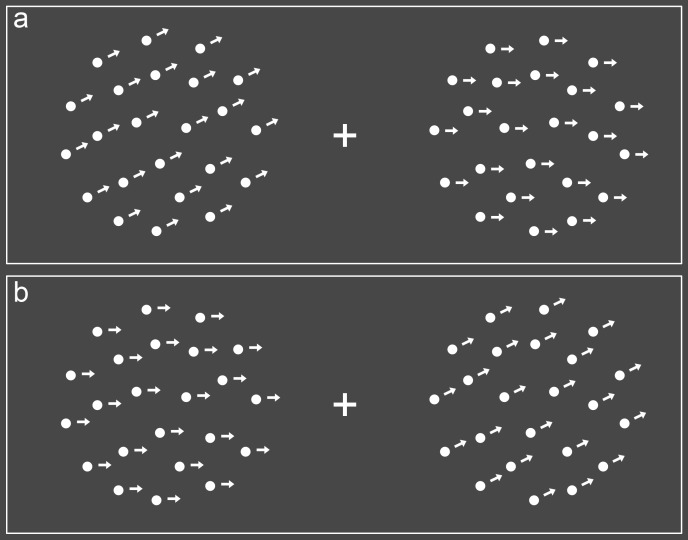
Stimuli in the direction of motion task. Trials consisted of diagonal motion on the left and horizontal motion on the right side (Panel A) or vice versa (Panel B). Arrows indicate the direction of motion for illustration purposes only. In this figure, diagonal and horizontal motions differ by an angle of 30°, the widest angle used.

Stimuli were the same as those in the movement localization task except for the following changes: The dots in each pattern were moving coherently and always to the right side with a speed of 0.15°/s. In half of the trials, dots on the left side were moving diagonal upwards whereas in the remaining trials, dots on the right side were moving diagonal upwards (see Figure 3). The dot pattern on the opposite side was always moving horizontally. The diagonal movement was presented at five different angles (with reference to the horizontal line), namely 3°, 6°, 9°, 20°, and 30°, as determined on pilot data. The condition with a divergence of 3° was therefore the least distinct.

#### Procedure

The procedure was identical to that of the movement localization task in all but the following aspects: Participants were instructed to indicate which of the two stimuli was moving diagonally by pressing either the left or right button, and to respond as fast and as accurately as possible, but to guess if they did not know the correct response. In order to maintain motivation of the participants, we also included wide angels (20° and 30°) that were easy to discriminate. By doing this, we could also double-check whether participants had followed the task instructions, as accuracy with the wide angles should be relatively high. In sum there were 300 trials, with a break after each 100 trials. Initially, participants completed 20 practice trials containing stimuli from the most distinct condition only (30° diagonal motion). Afterwards, they performed another 20 practice trials with stimuli from all conditions in random order. Participants who still did not feel comfortable with the task had the chance to do a further 20 mixed practice trials.

#### Data analysis

Median RTs were calculated as in the movement localization task. For the analysis of accuracy, trials without any response were excluded. Mean accuracies were calculated for each participant in each condition. These data were entered separately into two-way mixed ANOVAs, with the repeated-measures factor Angle (3°, 6°, 9°, 20°, 30°) and the between-subject factor Group (deaf, hearing). The factor Side was not included in these analyses since the direction of motion was not symmetrical. The dot patterns moved always to the right side. Greenhouse-Geisser corrected F and *p* values are reported in cases of violations of the sphericity assumption, and post hoc *t*-tests were performed where appropriate.

### Results

#### Response time

As expected, a significant main effect of angle, *F*(1.33, 34.50) = 51.75, *p* < .001, indicated that RTs decreased with increasing angle (paired samples *t*-tests for 3°: *M* = 844 ms, 6°: *M* = 788 ms, 9°: *M* = 722 ms, 20°: *M* = 609 ms, 30°: *M* = 593 ms; all *ps* < .01). This result is in line with a previous study investigating the ability to detect a change in direction of motion (Genova, Mateeff, Bonnet, & Hohnsbein, 2000).

**Figure 4.**
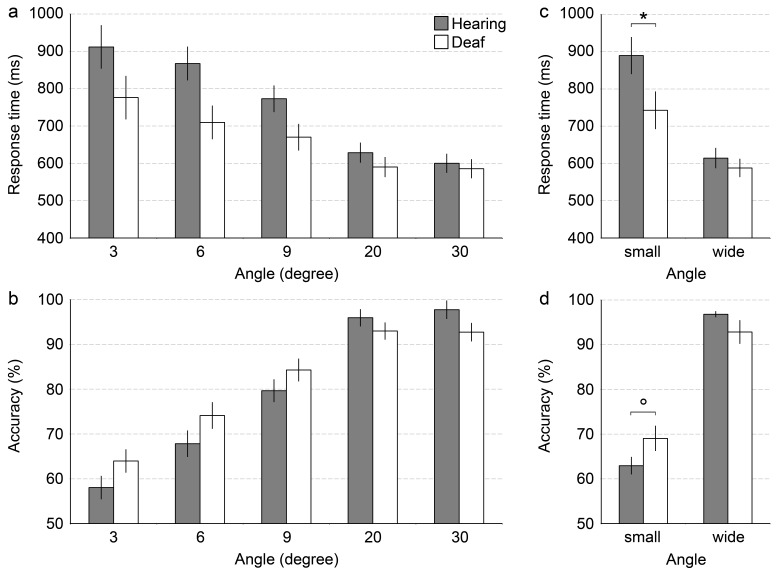
Grand mean of the participants’ median response times in milliseconds (Panel A) and mean accuracies in per cent (Panel B) in the direction of motion task for each angle and each group. Panels C and D show data averaged across angles for the small (3° and 6°) and wide (20° and 30°) angle conditions for response times and accuracies, respectively. Error bars represent between-subject standard errors of the mean (SEM). *p < .05. °*p* < .10.

The interaction between Angle and Group reached significance, *F*(1.33, 34.50) = 4.10, *p* < .05 (see Figure 4, Panel A). To reduce the number of comparisons for follow-up *t*-tests, RTs for the two easiest angles (20° and 30°) were averaged, constituting a wide angle condition whereas the mean of the RTs for the two most difficult angles (3° and 6°) were averaged, constituting a small angle condition. For small angles, deaf participants responded faster than their hearing counterparts (*M* = 743vs. 890 ms), *t*(26) = 2.11, *p* < .05. No significant group difference was observed for the wide angles (*M* = 588 vs. 614 ms), *t*(26) = 0.75, *p* = .46

#### Accuracy

A significant main effect of angle, *F*(2.39, 62.09) = 130.08, *p* < .001, confirmed the intended task manipulation. Accuracies increased with increasing angle (3°: *M* = 61.0%, 6°: *M* = 71.0%, 9°: *M* = 82.0%, 20°: *M* = 94.5%, 30°: *M* = 95.2%, all *ps* < .001, except for the comparison of angle 20° with 30°: *p* = .20).

The interaction between Angle and Group was significant, *F*(2.39, 62.09) = 4.20, *p* < .05 (see Panel B of Figure 4). For subsequent independent *t*-tests between groups, data were again collapsed to build a wide angle (20° and 30°) and a small angle condition (3° and 6°). For small angles, deaf participants showed a trend towards more accurate responses than their hearing counterparts (*M* = 69.1 vs. 62.9%), *t*(26) = 1.84, *p* = .08. No significant group difference was observed for the wide angles (*M* = 92.9 vs. 96.8%), *t*(26) = 1.51, *p* = .15.

Overall, participants responded slower and tended to respond less accurately with decreasing angle of motion. Of interest in the present study, however, is that although there was no difference between deaf and hearing participants in their responses when stimuli differed by a wide angle, differences became evident with small (and therefore more difficult to distinguish) angles. Deaf participants responded faster and tended to be more accurate than hearing controls when small deviations in motion from the horizontal were to be detected. This finding will be further discussed below.

## General discussion

The present study compared visual motion perception in profoundly deaf and hearing humans. Comparable with a recent animal study (Lomber et al., 2010), participants completed a movement localization task and a direction of motion task. Overall, there was no difference in RTs between deaf and hearing participants in the movement localization task, although in the deaf group, a left visual field advantage was found. When discriminating the direction of motion, however, deaf participants responded faster and tended to be more accurate when detecting small differences in direction compared with the hearing controls.

### Visual movement localization in deaf individuals

The lack of any overall difference in RTs between deaf and hearing participants in the movement localization task was unexpected. This result contradicts the findings of Lomber et al. (2010) who showed superior performance in deaf compared to hearing cats using a movement detection task. In addition to fundamental differences between cats and humans in vision, there are several factors that might potentially contribute to these differing results. Whereas Lomber et al. (2010) measured accuracy thresholds, in the present experiment RTs at fixed speed levels were used as the dependent variable. The high accuracy at all speed levels in the present study could have contributed to the lack of group differences in RTs. Thus, if deaf individuals are indeed better at detecting motion this might have become evident with stimuli that were more difficult to detect than the moving dot patterns. A further difference is the eccentricity of the visual dot patterns, which were presented at a larger eccentricity by Lomber et al. (2010) compared to the present study. However, it seems unlikely that this latter detail is the crucial difference, as deaf humans have been shown to be faster than hearing controls at stimulus detection in both the parafoveal (i.e., close to central) and the more peripheral visual field (Bottari, Caclin, Giard, & Pavani, 2011; Bottari et al., 2010).

The present study showed a localization advantage for movement presented in the left visual field for the deaf participants. Note here that participants were instructed to fixate the centre of the screen during the whole experiment. Indeed participants needed to monitor both sides simultaneously, as stimuli were presented on both the left and the right. However, since there was no objective assessment of eye movements the visual field asymmetry in the present study should be interpreted carefully. The left visual field advantage reported here is in contrast to several previous studies that have reported an advantage for movement perception in the right visual field (Bosworth & Dobkins, 1999, 2002; Brozinsky & Bavelier, 2004; Neville & Lawson, 1987). Movements of the hands and of the upper part of the whole body are some of the main elements of sign language and some aspects of sign language processing are lateralized to the left hemisphere (for a critical review, see Campbell, MacSweeney, & Waters, 2008). Neville and Lawson (1987) proposed that the involvement of motion in sign language may play a role regarding the greater involvement of the left hemisphere in motion perception leading to a right visual field advantage in deaf individuals. The authors hypothesised that early language experience influences functional asymmetries between the hemispheres. Interestingly, participants of the studies that found a right visual field advantage acquired sign language before the age of 6 either from their deaf parents or in (pre-) school institutions (Bosworth & Dobkins, 1999, 2002; Brozinsky & Bavelier, 2004), and/or had at least one deaf relative implying exposure to sign language at an early age (Neville & Lawson, 1987). The bulk of deaf participants in the present study (13 out of 19) in which we contrarily report a left visual field advantage in motion processing acquired sign language rather late when they entered school (age 6 to 8) or even later. Thus, one could speculate that the starting point of sign language acquisition might play an important role in the development of functional hemispheric asymmeries.

Another possible explanation for the left visual field advantage reported here is based on previous neuroimaging studies presenting moving stimuli to deaf individuals. These have reported visual activation of the auditory cortex specifically in the right hemisphere (Fine et al., 2005; Finney, Clementz, Hickok, & Dobkins, 2003; Finney et al., 2001). The authors suggested that the observed right-hemisphere preponderance for functional changes might reflect a predisposition of the right auditory cortex for motion processing. It was argued that the deprived auditory cortex, which is normally involved in the processing of auditory motion (Baumgart, Gaschler-Markefski, Woldorff, Heinze, & Scheich, 1999; but see also Smith, Okada, Saberi, & Hickok, 2004) may change its function towards the processing of visual motion. Against the background of the right-hemisphere preponderance for functional changes and the contra lateral projection of the visual pathway, and despite our note of caution above, a left visual field advantage for visual motion processing in deaf individuals appears to be a well-founded consequence of auditory deprivation. Further research employing objective fixation control is needed to support this interpretation.

### Direction of motion discrimination in deaf individuals

In the direction of motion task, for small angles, deaf participants responded faster and tended to perform more accurately than hearing controls. This is in line with results of Neville and Lawson (1987) who found deaf humans better able to indicate the direction of motion with greater attention-related visual evoked potentials in these participants. In contrast, both human (Bosworth & Dobkins, 1999, 2002) and animal (Lomber et al., 2010) studies reported that accuracy thresholds for direction of motion were similar in deaf and hearing individuals. In the present study, motion was presented with a slower velocity compared to these studies, and task demands in motion tasks are known to be affected by velocity. For example, Genova et al. (2000) reported that participants’ RTs for detecting a change in direction of motion increased with decreasing speed of motion. Thus, one could reason that compared to previous studies (Bosworth & Dobkins, 1999, 2002; Lomber et al., 2010) task demands were rather high for our direction of motion task in which deaf participants outperformed hearing controls. We speculate therefore that differences in task demands may play a crucial role in the degree to which deaf humans show behavioural advantages over hearing controls. Please note that motion stimuli in the present study represented first-order motion. Thus, a generalization to all kinds of motion stimuli (including second-order motion) should not be made.

### Explanations for an improved motion perception in deaf individuals

The advantage in detecting small deviations from horizontal movements for deaf compared to hearing participants constitutes a behavioural effect. These superior visual abilities in deaf participants can be considered as additional evidence that deaf individuals compensate for the loss of audition through a more effective use of their remaining senses (for a recent review, see Merabet & Pascual-Leone, 2010). Improved visual abilities in deaf compared to hearing individuals could originate from changes in visual cortex function after early onset deafness. Consistent with this view, deaf individuals have been found to show greater activation of motion selective areas including the medial temporal (MT) and medial superior temporal (MST) area when attending to randomly moving dot patterns in the visual periphery(Bavelier et al., 2000, 2001). Alternatively, or perhaps additionally, superior visual abilities in deaf individuals might occur due to a reorganisation of the auditory cortex towards visual processing. In accordance with this, animal studies have provided evidence for a causal relationship between visual activation of auditory cortex and visual task performance (Lomber et al., 2010). Although Lomber et al. did not find performance changes in a direction of motion task by deactivating the auditory cortex of deaf cats, a contribution of the auditory cortex in more demanding direction of motion tasks like the one used in the present study cannot be excluded.

Previous studies have suggested that supramodal skills shared across senses may be more likely to engage cross-modal plasticity as compared to sensory modality-specific skills (Lomber et al., 2010). In the present study, enhanced performance of deaf humans in the direction of motion task was observed. The discrimination of motion direction is an ability that one might need in the visual, auditory, and tactile modalities, and hence it can be considered supramodal in character (Bavelier & Hirshorn, 2010). Because the localization of motion is also suggested to be a supramodal function, however, one would expect similar performance advantages in deaf humans for both tasks in the present study; that is, movement localization and discrimination of motion direction. Interestingly, Lomber et al. (2010) reported enhancements for movement detection in deaf cats while discrimination of motion direction was similar between deaf and hearing cats. Thus, although cross-modal plasticity seems to be a relevant concept, supramodality alone cannot explain the difference in the results for the movement localization and the direction of motion tasks in the present study.

### Summary and conclusion

The present study aimed at better understanding changes in visual motion perception after early onset deafness. Deaf participants did not outperform hearing controls in movement localization, but did show enhanced performance when detecting small deviations from the horizontal in the angle of motion. These results extend previous findings concerning changes in visual movement perception as a consequence of auditory deprivation. In particular, our findings show that enhancements of visual movement perception in deaf individuals are not widespread but selective and are revealed only under specific conditions.
